# Effective respiratory management of asthma and COPD and the environmental impacts of inhalers

**DOI:** 10.1038/s41533-023-00346-7

**Published:** 2023-07-01

**Authors:** Omar S. Usmani, Mark L. Levy

**Affiliations:** 1 National Heart and Lung Institute, Imperial College London and Royal Brompton Hospital and St Mary’s Hospital, London, UK; 2 Locum General Practitioner, London, UK

**Keywords:** Patient education, Quality of life, Public health, Disease prevention

Over the past few years, multiple health agencies from across the globe have recommended schemes to reduce the carbon footprint of inhalers, encouraging a switch to environmentally friendly alternatives. To control emissions from fluorinated greenhouse gases (F-gases), including hydrofluorocarbons (HFCs), the European Union has adopted the F-gas regulation^1^. Moreover, there is a drive in the UK to reduce the prescribing of pressurised metered-dose inhalers in favour of devices with lower global warming potential^2^. This initiative is in the context of poor asthma outcomes in the UK, particularly with deaths being among the highest in the world^3,4^. Furthermore, asthma care in the UK may be delegated to professionals without adequate training. According to the UK National Review of Asthma Deaths, 46% of nurses who had performed reviews on patients who died from asthma had no formal training in asthma care. Moreover, sometimes those performing asthma reviews did not recognise patients who were at risk, which could have resulted in potentially preventable deaths^5^. Thus, we emphasise that the optimal management and quality of care of patients with asthma and chronic obstructive pulmonary disease (COPD) needs to remain the absolute priority for prescribers, to avoid exacerbating outcomes further. Selecting the inhaler device best suited to an individual patient is a challenging process, with many contributing factors. Among these, to reduce the environmental impact of inhalers we believe that it is important to consider correct use^6,7^, adherence^8^ and acceptability^9^ of prescribed devices. The most appropriate and environmentally friendly inhaler is one that a patient will adhere to and use correctly: this minimises wastage and promotes good disease control. Changing a patient’s prescribed inhaler when their condition is stable on their current device risks loss of disease control and is, in our view, clinically irresponsible. If a change is clinically warranted, it must be accompanied with a face-to-face consultation to ensure good inhaler technique and a comprehensive follow-up to confirm sustained clinical control of the disease. This article discusses the importance of combining effective respiratory management of asthma and COPD with environmental considerations (Box 1), and focuses on the UK as an example owing to the distribution of inhaler sales and poor patient outcomes.

## Box 1

Key messages for effective management of asthma and COPD, and the environmental impact of inhalers

- Environmental issues are important
- Patient care and reducing poor outcomes should be prioritised
- Switching patients to an environmentally friendly inhaler that they cannot use or do not like is counterproductive
  - Treatment of asthma attacks adversely affects the environment

## The prescriber’s perspective: overview

Recent reflections on the environmental impact of inhalers have led to discussions among physicians, patient groups and patients regarding ethical decisions in treatment choice for inhaled therapies. The headlines in mainstream media that asthma inhalers can ‘emit as much carbon as a 180-mile car journey’^10^ not only fail to highlight the priority of ensuring continued, optimal treatment for patients, but also risk stigmatising patients with asthma and COPD; this could lead to patients stopping or inappropriately switching inhalers and to a deterioration in outcomes. Emotive headlines aside, the environmental impact of inhaler devices merits consideration and is now, somewhat understandably, being reflected in health policy decisions. These include recommendations from multiple health agencies across the globe to implement schemes to reduce the carbon footprint of inhalers by encouraging a switch to environmentally friendly alternatives, such as the EU regulation on F-gases and the National Health Service (NHS) net zero commitment in the UK^1,2^. Appropriate assessment of clinical need, the associated environmental impacts throughout the treatment pathway and the life cycle of the inhalers (including manufacturing, supply, use and disposal) is vital^11^. As prescribers, however, we have a responsibility to ensure that initiatives to reduce pollution do not have detrimental effects on patients’ health. Our priority should remain to prescribe the right medication for the patient in an inhaler device that they like and can use properly and effectively^9^. It is vital that we, and particularly patients, recognise the difference between modifiable lifestyle factors (such as driving a car) and a requirement for effective healthcare. Over the coming years, we will see the introduction of propellants for pressurised metered-dose inhalers (pMDIs) with lower carbon footprints^12^. Hopefully, there will also be increased efforts towards improved recycling facilities for inhalers.

Globally, 97% of all reliever inhaler prescriptions are pMDIs^13^; however, there is a significant variability in the proportion of inhaler doses (excluding nebulised) given by pMDI, which ranges from 34% in Japan to 88% in the USA^14^. In Europe, the most common reliever inhalers sold in 2011 were pMDIs (47.5%) followed by dry-powder inhalers (DPIs; 39.5%) and nebulisers (13%)^15^. Nonetheless, the prevalence of inhaler prescriptions by device category in Europe varies substantially by country^16^, with pMDIs accounting for a significant proportion (60–70%) of inhaler use in the UK^6^, compared with just 13% in Sweden^17^. Implementation of European and UK policies aimed at reducing prescription of pMDIs in favour of devices with lower global warming potential^1,2^ may have negative effects on patient outcomes and undesirable adverse consequences for patient health^13^, particularly in countries where pMDIs are most frequently prescribed, such as the UK^16^. Even in countries where the proportion of DPI use is high, short-acting β2-agonists (SABAs) are significantly relied upon, which may indicate poor asthma control and adverse health outcomes^18^. This emphasises that education and support are crucial to achieve correct device usage^15^, regardless of inhaler type, and that a change of device should prioritise the improvement of symptoms and an increase in patient compliance and convenience^8^.

## Situation in the UK

The quality of patient care needs to be the absolute priority in driving prescribing trends. Asthma outcomes in the UK are currently among the worst in the world: childhood asthma deaths are the highest in Europe and the fourth highest in the world among 10–24-year-olds^3,4^. It is of critical importance to improve health outcomes while taking into account environmental considerations.

In the UK, the use of pMDIs versus DPIs is high compared with other European countries: a previous analysis of device prescriptions in 16 European countries (2002–2008) revealed that the UK had the highest rate of pMDI sales (approximately 70%, compared with <50% in the other European countries studied)^6,16^. Concerns have been raised regarding the environmental impact of pMDIs, which contain propellant gases that have a high global warming potential (GWP). Propellants based on chlorofluorocarbons were banned many years ago, but the hydrofluoroalkane (HFA)-based propellants adopted in their place also have a carbon footprint, although much lower. One study by GlaxoSmithKline reported that the GWP of hydrofluorocarbon (HFC)-134a-containing pMDIs was 17 times higher than that of DPIs^19^; however, this focused only on GWP, and did not consider the full environmental impact of DPIs throughout their life cycle, such as toxicity to humans, marine eutrophication and fossil depletion^12^. A study of UK NHS prescription records in 2017 collated carbon footprint data of inhalers commonly used in England and suggested that switching from pMDIs to DPIs could provide large carbon savings and reductions in drug costs, if less expensive brands are also used^20^. However, it should be emphasised that this study did not consider the potentially devastating unintended consequences of interrupted and/or suboptimal disease control. These could include poor patient outcomes, acute care costs and the associated carbon footprint of avoidable emergency and in-hospital visits and management^11,21^. A recent study on greenhouse gas emissions linked to asthma control in the UK reported that 63% of the carbon footprint associated with asthma medication is attributable to reliance on SABA use^22^. By ensuring that all people with asthma are prescribed an inhaled corticosteroid (ideally as a ‘maintenance and reliever therapy’ regimen for as-needed relief) and limiting the prescription of SABAs to one per year for emergency use only, there could be a substantial reduction in the numbers of asthma attacks (and therefore hospital admissions) and of pMDIs prescribed in the UK^23–28^. Thus, a shift in UK practice towards reducing over-prescribing of SABA medications for asthma management would offer both clinical and environmental benefits^29^.

Inhaler technique training with a healthcare professional plays a crucial but undervalued role in patient outcomes, and the fact that inhaler technique remains poor with little sign of improvement suggests a failure of care; indeed, inhaler errors are associated with poor disease outcomes and greater health-economic burden^7^. Evidence suggests that before switching to a different inhaler, prescribers should consider training patients on the correct use of their current device^7^. Given that technique training has a major impact on patient outcomes, there is a need to ensure that patients are fully educated on the correct use of inhalers^30^.

pMDIs have been shown to make a small contribution (<0.1%) to total global emissions^31^. In the UK, approximately 3% of the NHS carbon footprint is attributable to inhaler devices^2^. Reducing emissions from this group of medicines is a target of the NHS net zero commitment, which encourages increased prescribing of DPIs over pMDIs (if clinically appropriate and done as an outcome of shared decision-making)^2,32^. According to the NHS Long-Term Plan, this can be achieved alongside increasing the capacity for the greener disposal of used inhalers, although this remains to be put in place^32^. It is vital that we urge caution among NHS managers and the clinical community regarding the potential risks of switching inhaled medications when not clinically warranted or without appropriate support and monitoring, as per the considerations included in recent NHS guidance^32^.

## Choosing an inhaler

There is a wide variety of drug combinations and inhaler devices for the management of asthma and COPD, and this diversity enhances patient and clinician choice. In Europe, there were more than 230 different device-drug combinations in 2011, with 48 different inhaler products available to prescribers in the UK alone^33^. Unfortunately, not all clinicians are familiar with the correct techniques for teaching about and using these devices, resulting in some patients not receiving adequate instructions^30^. Real-world evidence has established the importance of patient satisfaction with their inhaler, irrespective of the medication it delivers, in promoting adherence and good asthma control^9^. Several patient-, medication- and device-related variables contribute to determining the most appropriate treatment for an individual patient^15^, and the benefits of individualised training in device handling are testament to the complexities of promoting good adherence in this field^34^.

For a patient whose asthma or COPD is stable with their current inhaler, switching their medication for a non-medical reason is, in our view, unjustifiable and clinically irresponsible. Introducing a new device type without ensuring that the patient can use the device, or switching a patient’s prescribed inhaler when their condition is stable on their current device may lead to potential consequences resulting in disease destabilisation, which is associated with considerable patient harm and unnecessary acute services costs^21^. Moreover, it can have variable clinical consequences^35^, because the patient may struggle to adapt to a different inhalation technique or may simply take less medication than prescribed because of dissatisfaction with their new device. Critical errors in inhaler handling are common and associated with poor disease outcomes^7^. Thus, any switch to a new device should be supported by a face-to-face consultation by someone with appropriate training, plus subsequent review appointments after the switch, as proposed in the ‘assess, adjust and review’ cycle in the 2022 Global Initiative for Asthma (GINA) recommendations^27^; all of these approaches require appropriately trained healthcare professionals and resources, resulting in additional time and costs^8^. Switching inhaler may not be an option for many patients. For example, DPIs are not appropriate for younger children, and the need for a particular medication may restrict device choice^14^. Scenario analyses using asthma and COPD inhaler usage data from 2019 indicate that a strategy in which pMDIs are transitioned to use a low-GWP propellant (HFA-152a) is a more favourable option than the substitution of pMDIs with DPIs or soft-mist inhalers, while also preserving patient access and choice^31^. Changing the type of propellant instead of the type of inhaler may provide an alternative solution to reduce the GWP of inhaler usage, especially for patients who prefer pMDIs and for whom this type of inhaler is the most clinically appropriate^31^. On a wider scale, some countries are largely dependent on pMDIs, owing to the higher cost of DPIs^14^. Thus, pressures to reduce the carbon footprint by switching to low-GWP products in pMDIs may have a substantial impact on costs for low- and medium-income countries that mainly rely on pMDIs, especially considering that low-GWP propellants are manufactured by Western countries^14^.

Experience of policies supporting changes in prescribing habits for cost-saving purposes suggests that switching inhalers on non-medical grounds leads to reduced adherence, loss of disease control and a subsequent increased demand for healthcare resources and incurred costs^8^. Similarly, the indirect impact of reduced disease control on the environmental burden needs to be considered very carefully: increased hospitalisations and rescue medication use, together with wasted medication, are all likely consequences of an ill-advised medication switch and are associated with increased contributions to the carbon footprint. A study in patients with asthma enrolled in the Clinical Practice Research Datalink (2007–2017) estimated that 1 year’s worth of greenhouse gas is emitted from medications, exacerbations and healthcare resource utilisation, and reported a notably smaller overall environmental burden with controlled than with uncontrolled asthma^22^. Another recent work highlighted that a history of severe or multiple COPD exacerbations can increase the carbon footprint of future healthcare resource utilisation and SABA prescribing by 50% for each year of follow-up^36^. Interestingly, a recent report analysing NHS England’s greenhouse gas emissions based on clinical activity for 2019 has shown that health care provided by acute services is the largest contributor of total emissions, comprising more than half (56%) of these; furthermore, the impact on GWP associated with the delivery of acute services is greater than that associated with HFC-containing pMDIs^37^. This evidence reinforces the argument that optimal disease control is the most appropriate target, both for quality of patient care and to minimise environmental burden.

## The need to reduce the environmental burden of inhalers

Although we are clear that optimising patient care should be prioritised when considering whether to change treatments, the impact of inhaler devices on the environment is going to remain a concern. While efficacy and safety of treatments must be the priority, in recent years the environmental impact of inhalers has become an increasingly necessary consideration. We believe that if an equivalent is available, in terms of ease of use and deliverability of the drug, and if the patient can use this correctly and prefers to do so, the inhaler with the lowest environmental impact should be first choice. Nevertheless, it is important to focus not only on the GWP of inhalers, but also on the potential impact on terrestrial and marine welfare. To analyse the environmental impact of different types of inhaler throughout their life cycles, a study modelled seven different scenarios that may reduce the environmental impact of inhaler devices^12^. Replacing all pMDIs with DPIs was the least favourable strategy for eight of the 14 environmental impacts considered, with particularly detrimental effects on marine eutrophication, fossil fuel depletion and photochemical oxidant formation. Other strategies assessed in the model included changing the propellant contained in the pMDI and recovering propellant from used pMDI devices^12^.

## Improving patient care and reducing the environmental burden

A simple change in the recommended management of asthma could have a significant impact both on the levels of disease control achieved and the overall GWP of inhaler usage.

**Fig. 1:**
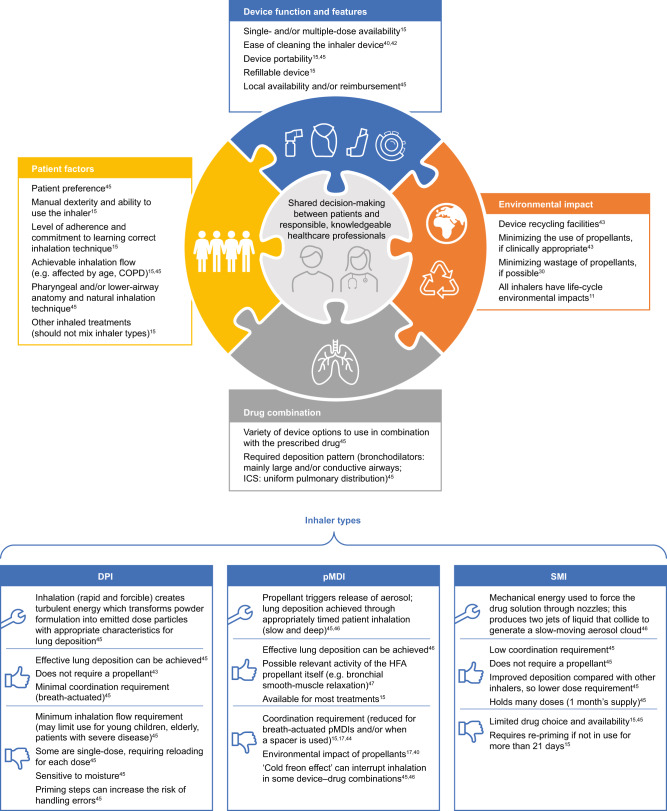
Considerations for selecting a particular inhaler device and drug combination^11,15,17,30,40,42–47^. COPD chronic obstructive pulmonary disease, DPI dry-powder inhaler, HFA hydrofluoroalkane, ICS inhaled corticosteroid, pMDI pressurised metered-dose inhaler, SMI soft-mist inhaler.

Of course, there are situations when switching an inhaled medication is opportune. Clearly, it is appropriate to consider the environmental impact when selecting an inhaler at treatment initiation or when stepping up or down treatment, but only after careful consideration of the patient’s needs, their preferences, their ability to use the inhaler and their response to treatment. In a time-limited consultation, clinical need (ensuring the most suitable inhaler choice for each patient) should be prioritised over a detailed review of the environmental issues. If the patient raises queries relating to environmental impact, it is important that the prescriber can discuss this topic objectively with the patient in greater detail than the basic difference in GWP of pMDIs versus DPIs. The patient should be offered a clear summary of the wider picture in terms of the overall life cycle of the various inhalers available to them (Fig. 1). For balance, patients should be made aware of any disposal and recycling initiatives that are available to them, as well as of forthcoming developments in propellant technology. The initiation of a nationwide recycling scheme would also require support from the government: currently, NHS England and NHS Improvement do not have any plans in place for national inhaler recycling schemes, but they encourage local and manufacturer-led inhaler disposal programmes. For example, the ‘Take Action for Inhaler Recycling’ pilot scheme, funded by Chiesi Limited, launched in Leicestershire^38,39^. It is vital that patients understand that achieving optimal disease control is the priority, both for their own personal well-being and for the wider implications of healthcare resource use and environmental impact.

In conclusion, the detrimental effect of inhalers on the environment has recently been highlighted in both the media and the scientific community^10,17,40^, and is beginning to be reflected in treatment strategies for asthma and COPD; however, this issue must be considered in the wider context of optimising the management of these diseases and with full engagement of patients. A switch in prescription without medical justification is inappropriate and unsafe. It may also have a net negative impact on the environment due to the additional healthcare resource use associated with loss of disease control. We believe that environmentally friendly inhalers are those that patients use correctly and according to the prescribed regimen. Any changes in treatment should be considered carefully to ensure that the pursuit of reducing the environmental impact of the life cycle of the inhaler does not restrict the patient’s choice or have unintended consequences for patient care. Innovation in inhaler devices, propellant technology and drug combinations are ongoing, along with developments to monitor and to improve inhaler technique and adherence^41^. These advances have the potential to reduce the environmental burden associated with inhaler use significantly in the future. As a more immediate and urgent strategy in the asthma setting, the priority should be to focus on improving outcomes for patients to levels similar to or above those of European countries and reducing the number of avoidable deaths from asthma. An approach that might be easily adopted could involve a detailed analysis of real-world data from practices in which the proportions of pMDIs or DPIs are particularly high, allowing us to understand the reasons underlying potential differences in patients’ clinical outcomes and to make informed decisions on inhalers that best match patients’ needs. Educating both prescribers and patients on the merits of minimising dependence on SABA medications and prioritising effective controller therapy will, in our view, improve disease outcomes and reduce the impact on the environment.

### Reporting summary

Further information on research design is available in the Nature Research Reporting Summary linked to this article.

## Supplementary information

Reporting Summary

## Data Availability

No data were generated in the preparation of this manuscript.

## References

[1] EU Legislation in Progress. Review of the regulation on fluorinated greenhouse gases. https://www.europarl.europa.eu/RegData/etudes/BRIE/2022/733673/EPRS_BRI(2022)733673_EN.pdf (2022).

[2] National Health Service. Delivering a ‘net zero’ National Health Service. https://www.england.nhs.uk/greenernhs/wp-content/uploads/sites/51/2020/10/delivering-a-net-zero-national-health-service.pdf (2020).

[3] Shah, R., Hagell, A. & Cheung, R. International comparisons of health and wellbeing in adolescence and early adulthood. Research report, Nuffield Trust and Association for Young People’s Health. https://www.nuffieldtrust.org.uk/research/international-comparisons-of-health-and-wellbeing-in-adolescence-and-early-adulthood (2019).

[4] Wolfe I (2013). Health services for children in western Europe. Lancet.

[5] National Review of Asthma Deaths. Why asthma still kills. https://www.rcplondon.ac.uk/projects/outputs/why-asthma-still-kills (2014).

[6] Attar-Zadeh D, Lewis H, Orlovic M (2021). Health-care resource requirements and potential financial consequences of an environmentally driven switch in respiratory inhaler use in England. J. Health Econ. Outcomes Res..

[7] Usmani OS (2018). Critical inhaler errors in asthma and COPD: a systematic review of impact on health outcomes. Respir. Res..

[8] Bjornsdottir US, Gizurarson S, Sabale U (2013). Potential negative consequences of non-consented switch of inhaled medications and devices in asthma patients. Int J. Clin. Pr..

[9] Plaza V (2018). Impact of patient satisfaction with his or her inhaler on adherence and asthma control. Allergy Asthma Proc..

[10] Matthews, S. Asthma inhalers emit as much carbon as a 180-mile car journey, says Government health watchdog as it urges patients to use more environmentally-friendly devices. *Daily Mail*. (2019). https://www.dailymail.co.uk/health/article-6899525/Asthma-patients-urged-consider-environmentally-friendly-inhalers.html (2022).

[11] Righton L (2021). Six inhaler sustainability myths—and why they must be busted. ONdrugDelivery.

[12] Jeswani HK, Azapagic A (2019). Life cycle environmental impacts of inhalers. J. Clean. Prod..

[13] Usmani OS, Scullion J, Keeley D (2019). Our planet or our patients—is the sky the limit for inhaler choice?. Lancet Respir. Med..

[14] Pritchard JN (2020). The climate is changing for metered-dose inhalers and action is needed. Drug Des. Dev. Ther..

[15] Usmani OS (2019). Choosing the right inhaler for your asthma or COPD patient. Ther. Clin. Risk Manag..

[16] Lavorini F (2011). Retail sales of inhalation devices in European countries: so much for a global policy. Respir. Med..

[17] Janson C (2020). Carbon footprint impact of the choice of inhalers for asthma and COPD. Thorax.

[18] Nwaru BI (2020). Overuse of short-acting beta(2)-agonists in asthma is associated with increased risk of exacerbation and mortality: a nationwide cohort study of the global SABINA programme. Eur. Respir. J..

[19] GlaxoSmithKline. Complete the cycle. http://uk.gsk.com/en-gb/responsibility/ourplanet/complete-the-cycle/ (2014).

[20] Wilkinson AJK, Braggins R, Steinbach I, Smith J (2019). Costs of switching to low global warming potential inhalers. An economic and carbon footprint analysis of NHS prescription data in England. BMJ Open.

[21] Levy, M. L. Inhaler devices and global warming: Flawed arguments. *BMJ Open* **9**, https://bmjopen.bmj.com/content/9/10/e028763.responses (2019).

[22] Wilkinson A (2021). Greenhouse gas emissions associated with asthma care in the UK: results from SABINA CARBON. Eur. Respir. J..

[23] Suissa S, Ernst P (2001). Inhaled corticosteroids: impact on asthma morbidity and mortality. J. Allergy Clin. Immunol..

[24] Suissa S, Ernst P, Benayoun S, Baltzan M, Cai B (2000). Low-dose inhaled corticosteroids and the prevention of death from asthma. N. Engl. J. Med..

[25] Suissa S (1994). A cohort analysis of excess mortality in asthma and the use of inhaled beta-agonists. Am. J. Respir. Crit. Care Med..

[26] Suissa S, Ernst P, Kezouh A (2002). Regular use of inhaled corticosteroids and the long term prevention of hospitalisation for asthma. Thorax.

[27] Global Initiative for Asthma (GINA). Global strategy for asthma management and prevention. https://ginasthma.org/wp-content/uploads/2022/05/GINA-Main-Report-2022-FINAL-22-05-03-WMS.pdf (2022).

[28] Cabrera CS (2020). SABINA: global programme to evaluate prescriptions and clinical outcomes related to short-acting beta2-agonist use in asthma. Eur. Respir. J..

[29] Scullion J (2020). Sustainability 3: how nurses can reduce the environmental impact of inhalers. Nursing Times [online].

[30] Keeley D, Scullion JE, Usmani OS (2020). Minimising the environmental impact of inhaled therapies: problems with policy on low carbon inhalers. Eur. Respir. J..

[31] Pernigotti D (2021). Reducing carbon footprint of inhalers: analysis of climate and clinical implications of different scenarios in five European countries. BMJ Open Respir. Res..

[32] National Health Service. Network Contract Directed Enhanced Service. Investment and Impact Fund 2022/23: Updated Guidance. March (2022).

[33] Rogueda P, Traini D (2016). The future of inhalers: how can we improve drug delivery in asthma and COPD?. Expert Rev. Respir. Med..

[34] Sanchez-Nieto JM (2022). Effectiveness of individualized inhaler technique training on low adherence (LowAd) in ambulatory patients with COPD and asthma. NPJ Prim. Care Respir. Med.

[35] Usmani, O. S. et al. Real world impact of non clinical inhaler switching on asthma or COPD patients—a systematic review. In: *American Thoracic Society International* (Virtual Congress, 2022).

[36] Bell J, Ding B, De Nigris E, Haughney J (2021). Greenhouse gas emissions associated with COPD care in the UK: Results from SHERLOCK CARBON. Eur. Respir. J..

[37] Tennison I (2021). Health care’s response to climate change: a carbon footprint assessment of the NHS in England. Lancet Planet Health.

[38] Robinson, J. NHS England has no plans for a national inhaler recycling scheme despite net zero ambitions. *Pharm. J.* **307** (2021).

[39] Murphy A, Howlett D, Gowson A, Lewis H (2023). Understanding the feasibility and environmental effectiveness of a pilot postal inhaler recovery and recycling scheme. NPJ Prim. Care Respir. Med.

[40] DeWeerdt S (2020). The environmental concerns driving another inhaler makeover. Nature.

[41] Bonini M, Usmani OS (2018). Novel methods for device and adherence monitoring in asthma. Curr. Opin. Pulm. Med.

[42] Asthma and Lung UK. Cleaning and looking after your inhaler. https://www.asthma.org.uk/advice/inhalers-medicines-treatments/inhalers-and-spacers/cleaning-and-looking-after-your-inhaler (2022).

[43] British Thoracic Society. Position statement: the environment and lung health 2020. https://www.brit-thoracic.org.uk/document-library/governance-and-policy-documents/position-statements/environment-and-lung-health-position-statement-2020/#:~:text=Our%20objectives%20are%20to%20develop,about%20prevention%20of%20respiratory%20diseases. (2019).

[44] Haughney J (2010). Choosing inhaler devices for people with asthma: current knowledge and outstanding research needs. Respir. Med..

[45] Newman SP (2005). Inhaler treatment options in COPD. Eur. Resp. Rev..

[46] Roche N, Dekhuijzen PN (2016). The evolution of pressurized metered-dose inhalers from early to modern devices. J. Aerosol Med. Pulm. Drug Deliv..

[47] Sellers WFS (2017). Asthma pressurised metered dose inhaler performance: propellant effect studies in delivery systems. Allergy Asthma Clin. Immunol..

